# Characteristics of Whale Müller Glia in Primary and Immortalized Cultures

**DOI:** 10.3389/fnins.2022.854278

**Published:** 2022-03-14

**Authors:** Xandra Pereiro, Sandra Beriain, Lara Rodriguez, David Roiz-Valle, Noelia Ruzafa, Elena Vecino

**Affiliations:** ^1^Experimental Ophthalmo-Biology Group, Department of Cell Biology and Histology, University of Basque Country UPV/EHU, Leioa, Spain; ^2^Begiker-Ophthalmology Research Group, BioCruces Health Research Institute, Cruces Hospital, Barakaldo, Spain; ^3^Department of Biochemistry and Molecular Biology, University Institute of Oncology (IUOPA), University of Oviedo, Oviedo, Spain

**Keywords:** Müller cells, glia, retina, whale, immortalization, cell line

## Abstract

Müller cells are the principal glial cells in the retina and they assume many of the functions carried out by astrocytes, oligodendrocytes and ependymal cells in other regions of the central nervous system. Müller cells express growth factors, neurotransmitter transporters and antioxidant agents that could fulfill important roles in preventing excitotoxic damage to retinal neurons. Vertebrate Müller cells are well-defined cells, characterized by a common set of features throughout the phylum. Nevertheless, several major differences have been observed among the Müller cells in distinct vertebrates, such as neurogenesis, the capacity to reprogram fish Müller glia to neurons. Here, the Müller glia of the largest adult mammal in the world, the whale, have been analyzed, and given the difficulties in obtaining cetacean cells for study, these whale glia were analyzed both in primary cultures and as immortalized whale Müller cells. After isolating the retina from the eye of a beached sei whale (*Balaenoptera borealis*), primary Müller cell cultures were established and once the cultures reached confluence, half of the cultures were immortalized with the simian virus 40 (SV40) large T-antigen commonly used to immortalize human cell lines. The primary cell cultures were grown until cells reached senescence. Expression of the principal molecular markers of Müller cells (GFAP, Vimentin and Glutamine synthetase) was studied in both primary and immortalized cells at each culture passage. Proliferation kinetics of the cells were analyzed by time-lapse microscopy: the time between divisions, the time that cells take to divide, and the proportion of dividing cells in the same field. The karyotypes of the primary and immortalized whale Müller cells were also characterized. Our results shown that W21M proliferate more rapidly and they have a stable karyotype. W21M cells display a heterogeneous cell morphology, less motility and a distinctive expression of some typical molecular markers of Müller cells, with an increase in dedifferentiation markers like α-SMA and β-III tubulin, while they preserve their GS expression depending on the culture passage. Here we also discuss the possible influence of the animal’s age and size on these cells, and on their senescence.

## Introduction

Müller glia are the predominant type of non-neuronal cells in the vertebrate retina, representing up to 90% of the retinal glia. The cell body of these glia spans the entire retinal depth, from the inner vitreal border to the outer limiting membrane (Ramón y Cajal, 1892; Reichenbach and Robinson, 1995; Bringmann et al., 2006). Aside from providing structural stability to the retina, Müller cells also take part in a multitude of retinal activities and they are essential to maintain retinal homeostasis. By maintaining close contact with the rest of the retinal cells, they establish metabolic partnerships with them and they constitute a common link between all retinal cell types. Müller cells are vital for neuronal survival and the formation of the inner blood-retina barrier (Bringmann et al., 2006; Vecino et al., 2016). However, not all Müller cells have the same characteristics, as it has been hypothesized that subpopulations of these cells may exist with distinct physical properties. This hypothesis is reinforced by the strong variation in molecular marker expression between Müller cells (Glial Fibrillary Acidic Protein-GFAP, glutamine synthetase -GS, class II major histocompatibility complex-MHC antigen, nestin, vimentin, and intermediate filament proteins) both in physiological and pathological conditions (Roberge et al., 1985; Luna et al., 2010; Vecino et al., 2016; Pereiro et al., 2020). Müller cells can also express neural markers such as β-III tubulin after retinal detachment (Lewis et al., 1995) and neural progenitor markers such as Pax6, Sox2 and nestin after retinal damage (Das et al., 2006; Xue et al., 2010; Joly et al., 2011). For instance, heterogeneous expression of GFAP and GS has been seen between individual cells in the same primary culture. These differences in expression were consistent in multiple species (pig, rat, and mouse), providing further evidence of the physiological heterogeneity among Müller cells in the retina. These characteristics could also affect cell-to-cell interactions and their activities (Pereiro et al., 2020).

Müller glial cells are capable of secreting neurotrophic factors that can contribute to both retinal neuron survival and axonal growth (Garcia et al., 2002; Ruzafa et al., 2018). Moreover, in teleost fish Müller cells display features of retinal stem cells, and they can proliferate and dedifferentiate into new cells, potentially serving as progenitor cells after retinal injury in adults (Bernardos et al., 2007; Otteson and Phillips, 2010; Rodriguez and Vecino, 2011; Nagashima et al., 2013). However, in the chick retina Müller cells can only fulfill this role during development and for a short period of time after hatching (Fischer and Reh, 2001). However, even though they can express stem cell related genes, the phenomenon of retinal regeneration and neurogenesis by Müller cells has not been described in mammals.

The physiological roles of Müller cells have been studied in primary cell cultures, examining the cellular, molecular, biochemical, electrophysiological and developmental features of these cells, together with the changes they undergo in pathological settings. However, primary cultures have some limitations, such as the limited life span of the cells in culture, the small number of cells unless many eyes are utilized, the difficulties in obtaining primary tissues from some species, etc. Some of these difficulties can be overcome by establishing immortalized cell lines, expanding the number of cells available and prolonging their life span (Sarthy et al., 1998). The immortalization of primary cells can be achieved by culturing them with viral oncogenes, such as the Simian Virus 40 (SV40). The SV40 genomic DNA encodes large and small T antigen (Tag) proteins, which drive SV40 transduced cells into the proliferation phase (Ahuja et al., 2005).

Multiple neuronal and glial cell types have been immortalized over the years, including Müller glia, generating cell lines that can be used for different purposes. Müller cell lines have been generated both by viral transformation and by spontaneous immortalization. The first such cell line was obtained in 1998, named rMC-1, a cell line derived from rat primary Müller cell cultures transformed with SV40 DNA. Conditionally immortalized cell lines are also of great interest, such as TR-MUL cell lines that possess a temperature-sensitive T antigen gene (Tomi et al., 2003). For example, the ImM10 murine Müller cell line was immortalized for two conditions simultaneously, temperature and activation by interferon-γ (IFN-γ) (Otteson and Phillips, 2010). A couple of spontaneously immortalized Müller cell lines have also been generated and it is generally considered that after multiple passages (20 passages or approximately 100 divisions), the cells that continue proliferating have overcome senescence and can be deemed immortal. In fact, the human Müller cell line MIO-M1 was generated this way (Limb et al., 2002) and a Müller cell line of murine origin, named QMMuC-1, also originated from spontaneous immortalization. Like MIO-M1 cells, the QMMuC-1 cells also preserve their Müller glial phenotype and characteristics, expressing lineage specific markers (Augustine et al., 2018). As well as using cells from adult retinas, cell lines originating from animals at different developmental stages have also been created, such as the spontaneously immortalized murine C57M10 Müller cell obtained from postnatal day (P10) mice (Otteson and Phillips, 2010). Müller cell proliferation kinetics has been studied in primary cells from a variety of species (pig, rat and mouse) (Pereiro et al., 2020). However, if immortalization affects the proliferation rates and kinetics of these cells and such changes have yet to be fully analyzed. Therefore, it is difficult to draw a clear relationship between primary cell and immortalized cell proliferation kinetics.

Although multiple Müller cell lines have been generated over the years, they have been derived from few species. Whales are the biggest mammals in the world, they live in an aqueous environment, hence, their physiology is very well adapted to withstand high pressures, for example, the elasticity of their lungs, their ribs which are held together by loose and flexible cartilage, allowing the rib cage to collapse at high pressures preventing the bones from breaking (Reidenberg, 2007). As far as the eye is concerned, the sclera is a thick, fairly hard structure that serves as a coffer in which sensitive parts of the eye such as the retina can be protected (Vecino et al., 2021). However, it is not known if the retina itself has their own adaptations to resist high pressures, so studying whale retinal cells could be of interest in relation to glaucoma, the second most important cause of blindness worldwide. While there could be much to be gained from studying whale Müller cells, whale specimens are not widely available. Therefore, the characterization of primary whale Müller cells and their immortalization would facilitate the study of the relationships between neurons and glia in these mammals. Few cetacean cell lines have been generated to date, one of which is a renal endothelial cell line derived from the bottlenose dolphin (Pine et al., 2004), and retinal or Müller cell lines from these mammals are not yet available.

Distinct cell lines of the same cell type have often been generated to study either differences between species, or to establish a platform to investigate specific factors or conditions in retinal health and disease. Here, we have cultured and immortalized whale (*Balaenoptera borealis*) Müller glia with SV40 T-antigen in order to examine the characteristics of these cells, as well as to study the proliferation kinetics of both primary and immortalized whale Müller cells. The cell line generated, named the Whale 2021 Müller (W21M) cell line, was studied by immunohistochemistry, scanning electron microscopy (SEM), karyotype characterization and time-lapse video analysis. Parameters related to proliferation were analyzed, including the time between divisions, the time Müller cells take to divide, the proportion of divisions, and the relative cell death and motility of the cells. W21M cells maintained their Müller glial phenotype, although they also presented characteristics of a partially dedifferentiated state in culture. The detailed analysis of their division kinetics and of other parameters allowed us to describe the changes in proliferation kinetics produced by immortalization in a cetacean model.

## Materials and Methods

### Study Design

In the present study, primary whale Müller cells were cultured, and some of these cells were immortalized with SV40 and compared to the primary Müller cells at different stages in culture.

### Whale Tissue and Culture

One eye from a beached Sei whale (*B. borealis*) was collected 24 h *post mortem* and once removed from the animal was maintained at 4°C. The whale retina was isolated and the Müller cells were cultured according to the protocols established previously (Pereiro et al., 2020). Briefly, an 8 mm diameter fragment of the whale retina was digested at 37°C for 30 min with papain (20 U/mL) and DNase (2,000 U/mL: Worthington) in Sterile Earle‘s Balanced Salt Solution (EBSS). This enzymatic digestion was stopped by adding Dulbecco’s Modified Eagle’s Medium (DMEM: Gibco Life Technologies) containing 10% Foetal Bovine Serum (FBS) and the retina was dissociated mechanically. The cell homogenate obtained was centrifuged at 1,200 rpm 5 min to remove debris and the pellet was resuspended in DMEM +10% FBS. The cells recovered were seeded at a density of 2.7 × 10^6^ cells in two 35 mm well plates coated with poly-L-Lysine (100 μg/ml: Sigma-Aldrich) and laminin (10 mg/ml: Sigma-Aldrich). The cell cultures were maintained in a humidified incubator at 37°C in an atmosphere of 5% CO_2_. The medium was changed on day 1 of the culture and half the volume of the medium was replaced every 2 days until the cultures were confluent. Pig and rat Müller cell cultures were performed following the same protocol. Once confluence was reached the culture was split 1:2, normally every 4–5 days.

### Immortalization

The primary Müller cells were immortalized using a lentivirus that expresses the large T-antigen of SV40, which interacts with and inhibits the p53 and retinoblastoma (Rb) tumor suppressors.

Lentivirus encapsulating the large T-antigen of SV40 were generated in HEK-293T human cells. Firstly, pLox-Ttag-iresTK plasmid (gift from Didier Trono, Addgene plasmid #12246) was transfected in these cells using Polyethyleneimine that is an amino-rich highly hydrophilic cationic polyelectrolyte that improve the transfection process by facilitating cellular endocytosis and protects plasmids from degradation (Lungwitz et al., 2005). After 24 h, the medium was replaced with fresh DMEM medium containing 10% FBS, and 24 h later the supernatant was collected and stored at −80°C for later use.

Immortalization commenced when the Müller cell culture reached 80% confluence. After 8 days *in vitro* (DIV), the lentiviral supernatants was diluted in DMEM and added to the cultured cells with polybrene (16 μg/ml), a cation polymer used to increase the efficiency of transduction of the cells was diluted in DMEM and added to the culture. After 24 h, the medium was replaced with fresh virus for a further 24 h and the transduced cells were then grown to confluence before they were sub-cultured. Parallel to the immortalization, other primary cell cultures were grown and split.

The cells were selected simply by culture passaging, growing primary and transduced Müller cells in parallel. At passage 8, the primary cells entered replicative senescence while the Tag transduced cells continued dividing and maintained a high level of viability. The cells that continued to undergo mitosis were considered to be immortalized Müller cells and they were cultured until passage 24.

### Karyotype

Primary cells at passage 7 and immortalized cells at passage 12 were incubated for 3.5 h in 0.01 μg/ml colcemid (KaryoMax, Gibco-Life Technologies), after which they were removed from the plates by standard trypsinisation and centrifuged in 12 ml conical test tubes for 5 min at 1,200 rpm. The cells recovered were then resuspended in a pre-warmed hypotonic solution (0.075 M KCl) for 20 min and fixed four times in methanol:acetic acid (3:1). Finally, the cells were spread on slides, air dried and aged for 4 days. After this period, chromosomes were G-banded and 10 metaphases from the immortalized cells were examined and scored for structural and numerical chromosomal aberrations.

At passage 7 there were not enough metaphases in the primary cell cultures to establish a clear karyotype since the cultures were reaching senescence.

### Whale Müller Cell Viability

The viability of immortalized Müller cells was measured until passage 11 and that of primary cells until passage 8. The same volume of trypan blue and cell suspension was mixed and loaded into a counting slice chamber. The live cell and total cell number was obtained on a TC20 automated cell counter (Bio-Rad), and the % cell viability was calculated as the ratio between the live and total cells. Finally, the % cell viability for primary and immortalized Müller cells was plotted against the passage number.

### Time-Lapse Recording and Analysis

**FIGURE 1:**
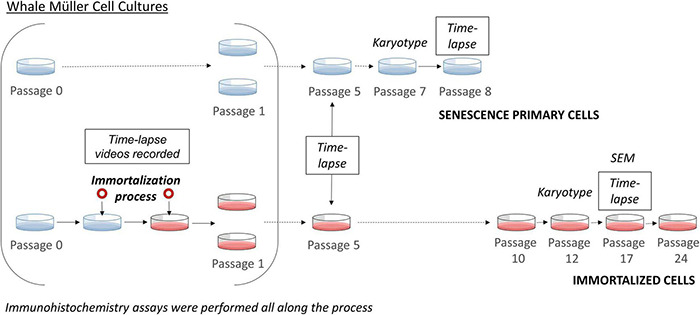
Scheme of the immortalization of whale Müller cells. Time-lapse imaging was performed during the immortalization process, at passage 5 for both primary and immortalized cells, at passage 8 of the primary cells and at passage 17 of the immortalized cell line. The karyotype of the primary cells was analyzed at passage 7 and that of the immortalized cell line at passage 12. At each passage immunocytochemistry analyses were performed.

The kinetics of whale Müller cells in culture was studied in time-lapse videos with one frame taken every 10 min for a minimum of 24 h. The videos were obtained at different time points or passages of the cell cultures and 10 scenes per video were analyzed (Figure 1). Specifically, time-lapse videos were recorded during immortalization (at passage 0, before, during and after SV40 large T-antigen transduction), at passage 5 of both the primary and immortalized Müller cells, at passage 8 of the primary cells, and finally, at passage 17 of immortalized cells given that the primary cultures reached senescence before that time. The videos of the cultures were recorded using a 20× objective in a Zeiss Axio Observer (Zeiss, Jena, Germany) coupled to a digital camera (Zeiss Axiocam MRM, Zeiss, Jena, Germany). During the video recordings, the cell cultures were maintained in a PM S1 incubator (Zeiss, Jena, Germany) at 37°C in a humidified atmosphere with 5% CO_2_.

The 10 scenes from each time-lapse video were analyzed manually to assess the alterations to Müller cell morphology, proliferation kinetics and cell death produced by immortalization. Between 30 and 120 cells were examined for each condition and for every parameter. To assess the proliferation kinetics of the immortalized Müller cell line, three parameters were monitored in each time-lapse video: (1) time between divisions, the time that each cell takes to divide again; (2) the time that the Müller cells take to divide, from cell rounding to cytokinesis; and (3) the percentage of dividing cells (proliferation).

To determine the time between divisions for each time point analyzed, the daughter cells generated after every cell division were followed manually until they themselves divided again. By following this procedure, the exact time (in hours) required for each cell to divide was determined. For each passage or time point, the mean of all the recorded division times was calculated. As for the length of division, the time (in minutes) that each cell needed to complete mitosis was assessed, from the point of cell rounding until cytokinesis occurred and the two daughter cells were evident, thereby ensuring that division had in fact occurred.

The proportion of dividing cells was calculated after the time between divisions was determined. The minimum time between divisions for each passage was used as the chosen time frame, and the number of divisions that occurred in that time frame were counted and divided by the number of cells at the start of the time frame. Similarly, the percentage of cell death was calculated by counting the number of dead cells and dividing this by the total number of cells at the start of the video, only recording the cell death detected on camera. For this, the cells were followed from when they still maintained a Müller cell morphology and could therefore be considered healthy, until they became rounded and their plasma membranes ruptured.

Finally, to measure the kinetics of cell motility, the average speed (μm/min) and the average distance covered (μm) were measured by tracking the position of the nucleus at every time frame using the CellTracker software (Piccinini et al., 2016).

### Immunocytochemistry

After every passage, the primary and immortalized Müller cells were analyzed by immunocytochemistry. The cells were washed in Phosphate Buffered Saline (PBS, pH 7.4), fixed in methanol at −20°C for 10 min and non-specific antigen binding was blocked with blocking buffer (0.1% Triton X-100 and 3% Bovine Serum Albumin -BSA- in PBS). The primary antibodies (see Supplementary Table 1) were diluted in blocking buffer and incubated with the cells overnight at 4°C. After washing three times in PBS, the cells were then incubated for 1 h at room temperature with the corresponding secondary antibodies diluted 1:1,000, Alexa Fluor 488 conjugated goat anti-mouse and Alexa Fluor 555 conjugated goat anti-rabbit antibodies (Invitrogen). After a further three washes, the coverslips were mounted with Fluor-save Reagent (Calbiochem) and the Müller cells were observed under an epifluorescence microscope (Zeiss Axiocam MRM, Zeiss).

Body and nucleus size were analyzed by ImageJ in immunostained pictures. Size was measured as area in μm^2^. For cell area the extent of vimentin was quantified and for nuclei size DAPI staining was measured. At least 20 cells from each animal were analyzed. Müller cells of all culture types were analyzed at 50% confluence in order to be able to count the cells separately. In addition, cells that were dividing or had just divided were avoided to measure. Results shown as Supplementary Figure 1.

### Scanning Electron Microscopy

Immortalized Müller cells were prefixed at passage 17 by adding 1.25% glutaraldehyde to the culture medium for 2 min and then further fixed with 2.5% glutaraldehyde for 15 min. The cells were washed three times in phosphate buffer (PB) and dehydrated for 10 min in a series of ethanol solutions: 30, 50, 70, 90, and 100%. Finally, the cells were incubated twice in hexamethyldisiloxane for 10 min and after drying overnight, the cell were gold-coated.

The samples were then put on the support and sputtered with a thin layer of gold under an argon atmosphere. They were visualized and photographed using Hitachi S-3400N scanning electron microscope with an accelerating voltage of 10 kV.

### Statistical Analysis

Statistical analyses were carried out using IBM SPSS Statistical software v.24-0, and the means and standard error of mean (SEM) are presented for each condition. The data from the different experimental conditions were compared using an analysis of variance (ANOVA), followed by the Bonferroni or Games–Howell test depending on the homogeneity of the variances. Differences were considered significant for all tests at *p* < 0.05.

## Results

### Karyotype

**FIGURE 2:**
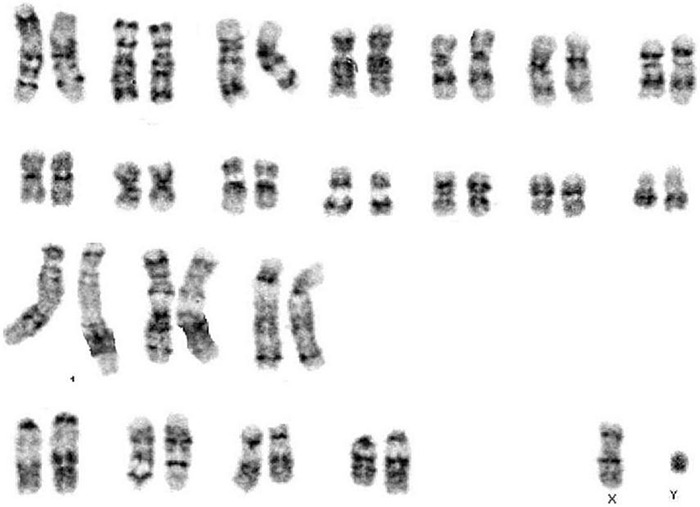
Karyotype from immortalized whale Müller cells at passage 12.

In order to validate the whale Müller cell line, we assessed the karyotype of these cells at passage 12. This karyotype did not show major aberrations and it conformed to a normal karyotype for the Sei whale: 2*n* = 44 chromosomes (Figure 2).

### Primary and Immortalized Müller Cell Viability

**FIGURE 3:**
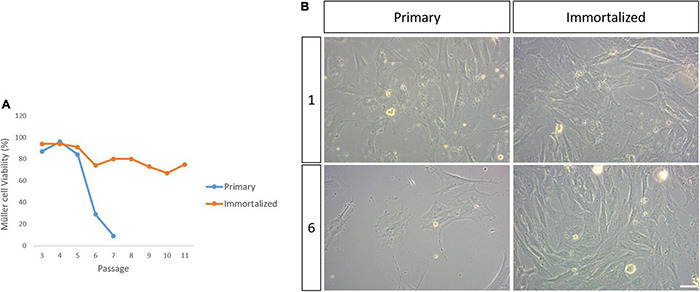
Viability of the primary and immortalized whale Müller cells in culture. **(A)** Plot of the percentage of viable primary and immortalized Müller cells at each passage. Note that the primary Müller cells reached senescence by passage 8 while the viability of the immortalized Müller cells remained high up to passage 11. **(B)** Images of the primary and immortalized whale Müller cell cultures at passage 1 and 6. There was a dramatic decrease in the number of viable primary Müller cells at passage 6. Scale bar: 50 μm.

The viability of the primary and immortalized Müller cells in culture was evaluated at each passage until passage 11 of the SV40 T-antigen transduced Müller cells, at which point they were considered to be immortalized. At passage 6, Müller cell viability in the primary cultures was 29%, decreasing from a viability of 55% at passage 5, and this drop in viability was more pronounced at passage 7 when only 9% of the cells were alive. The primary cell cultures were considered to have reached senescence at passage 8, however, the immortalized whale Müller cells maintained high levels of viability (94–75%) across all passages (Figure 3).

### Proliferation Kinetics

**FIGURE 4:**
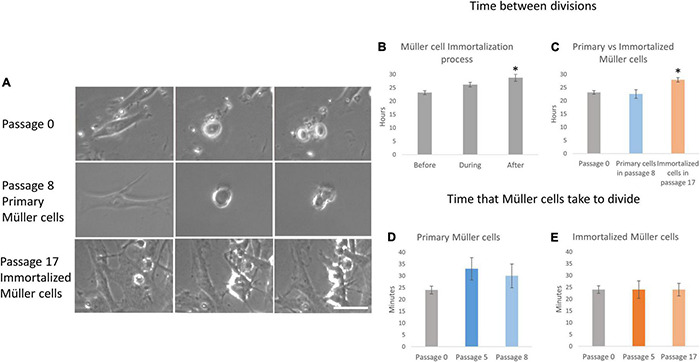
Proliferation kinetics of primary and immortalized whale Müller cells. **(A)** Sequence of 3 photos of a mitotic division of the whale Müller cell cultures at passage 0, of the primary Müller cells at passage 8 and of the immortalized Müller cells at passage 17. Note that in both primary Müller cells at passage 8 and immortalized cells at passage 17 it is harder to observe the cleavage furrow of cytokinesis relative to the cells at passage 0. **(B)** When the time between whale Müller cell divisions was analyzed during immortalization, it increased significantly immediately after immortalization. **(C)** The time between divisions was significantly longer in immortalized cells at passage 17 relative to passage 0. Analysis of the time that primary **(D)** and immortalized **(E)** whale Müller cells take to divide at different passages. Differences not observed: *p-value < 0.05, scale bar: 50 μm.

**FIGURE 5:**
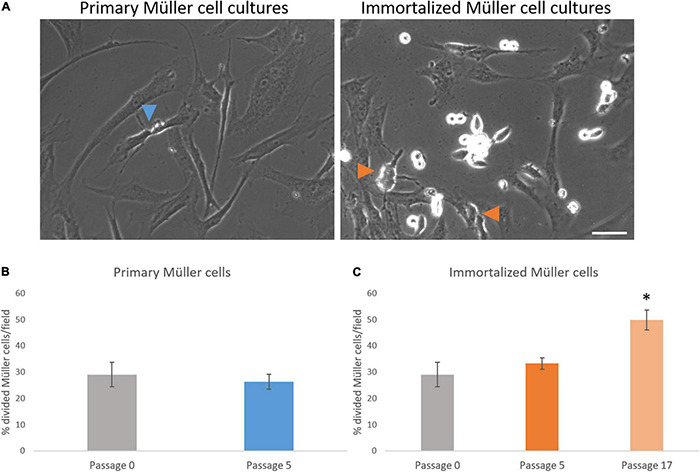
Analysis of the number of whale Müller cells that divided per field. **(A)** Images of primary and immortalized whale Müller cells in which the arrows indicate divisions. **(B)** Analysis of the percentage of cells that divided in primary cell cultures at passage 5. No differences were found when passage 5 was compared to passage 0. **(C)** Analysis of the percentage of cells that divided in immortalized whale Müller cells, which increased significantly at passage 17 compared to passage 0: *p-value < 0.05. Scale bar: 50 μm.

The behavior of primary and immortalized Müller cells in culture was also analyzed by time-lapse video, assessing the kinetics of proliferation, and quantifying the time between divisions, the time that Müller cells take to divide (Figure 4) and the number of Müller cells that divided per field (Figure 5). The time between divisions was analyzed during the immortalization of Müller cells (Figure 4B) and prior to immortalization this was 23.15 ± 0.71 h, increasing during immortalization to 24.35 ± 0.84 h and further immediately after immortalization to 28.71 ± 1.33 h (Table 1). Surprisingly, the time between divisions also increased significantly immediately after immortalization, as well as in subsequent passages of immortalized Müller cells, reaching 27.9 ± 0.86 h in immortalized Müller cells at passage 17 (Figure 4C and Table 1). The time that Müller cells take to divide (Figures 4D,E) was quantified, taking cell rounding as the starting point, and cytokinesis and the formation of two daughter cells as the final point. The time that primary Müller cells took to divide was 24 ± 1.63 min at passage 0, 33 ± 4.73 min at passage 5 and 30 ± 5 min at passage 8. Likewise, the time taken by immortalized Müller cells to divide was 24 ± 3.71 min at passage 5 and 24 ± 2.67 min at passage 17 (Table 2). Although significant differences were not observed in this analysis, cytokinesis at later passages was distinct to that at passage 0 in both primary and immortalized Müller cell cultures (Figure 4A).

**TABLE 1:** Time between divisions during immortalization and Time between divisions in primary and immortalized Müller cells.

Müller cells at passage 0	Time between divisions (h) during immortalization
Before immortalization	23.15 ± 0.71
During immortalization process	26.16 ± 0.84
After immortalization	28.71 ± 1.33

**Müller cells**	**Time between divisions (h) in primary and immortalized Müller cells**

Primary passage 0	23.15 ± 0.71
Primary passage 8	22.57 ± 1.65
Immortalized passage 17	27.90 ± 0.86

**TABLE 2:** Time taken by primary and immortalized whale Müller cells to divide.

Primary cells	Time takes to divide (min)	Immortalized	Time taken to divide (min)
Passage 0	24.00 ± 1.63	–	–
Passage 5	33.00 ± 4.73	Passage 5	24.00 ± 3.71
Passage 8	30.00 ± 5.00	Passage 17	24.00 ± 2.67

The number of Müller cells that divided per field was analyzed every 12 h, considering this as the shortest time between Müller cell divisions to avoid counting two divisions from the same cell. The percentage of primary Müller cells that divided at passage 0 (29.05 ± 4.60%) remained relatively constant at passage 5 (26.36 ± 2.81%), increasing at passage 8 (40.25 ± 6.95%). Similarly, the percentage of immortalized dividing Müller cells increased between passage 5 (33.28 ± 2.18%) and passage 17 (49.82 ± 3.86%: Table 3). Thus, in general the percentage of Müller cells that divided increased significantly from passage 0 to passage 17 (Figure 5).

**TABLE 3:** Percentage of primary and immortalized whale Müller cells dividing per field and percentage of cell death in primary and immortalized whale Müller cell cultures.

Primary cells	Percentage of dividing cells	Immortalized	Percentage of dividing cells
Passage 0	29.05 ± 4.60%	–	–
Passage 5	26.36 ± 2.81%	Passage 5	33.28 ± 2.81%
Passage 8	40.25 ± 6.95%	Passage 17	49.82 ± 3.86%

**Primary cells**	**Percentage cell death**	**Immortalized**	**Percentage cell death**

Passage 0	2.90 ± 0.27%	Passage 5	3.77 ± 0.36%
Passage 5	7.46 ± 0.80%	Passage 17	3.12 ± 0.61%

### Cell Death

**FIGURE 6:**
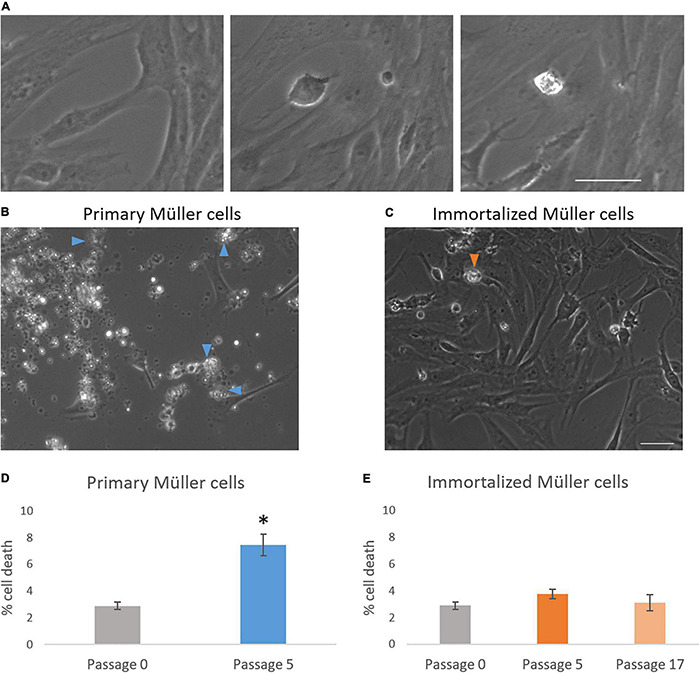
Analysis of cell death in primary and immortalized whale Müller cell cultures. **(A)** Chronological images of a Müller cell death. Images from primary **(B)** and immortalized **(C)** Müller cell cultures where arrows indicate dying Müller cells. **(D)** When the percentage of cell death was analyzed in primary Müller cell cultures at passage 0 and passage 5, cell death increased significantly at passage 5 of the culture. **(E)** When the percentage of cell death was analyzed in immortalized Müller cell cultures at passage 0, 5, and 17 no significant differences were found: *p-value < 0.05. Scale bar: 50 μm.

The time-lapse videos allowed cell death in the cultures to be studied. The percentage of dying cells in primary culture increased from passage 0 (2.90 ± 0.27%) to passage 5 (7.46 ± 0.80%), whereas the cell death in the immortalized cultures remained fairly constant from passage 5 (3.77 ± 0.36%) to passage 17 (3.12 ± 0.61%) (Table 3). As expected, cell death had increased significantly in primary Müller cell cultures at passage 5 (Figure 6).

### Motility Kinetics Analysis

**FIGURE 7:**
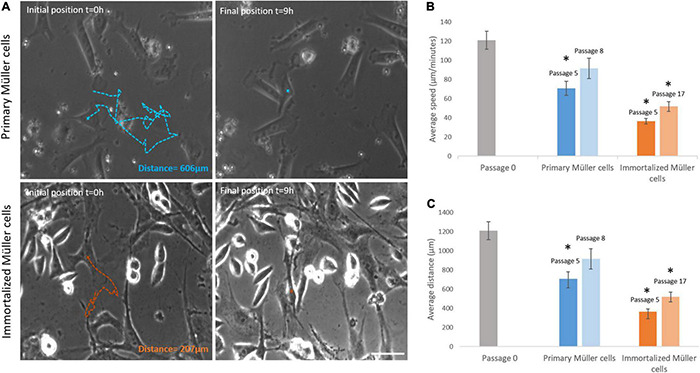
Primary and immortalized whale Müller cell motility. **(A)** Images of primary and immortalized cells tracked over 9 h. Initial position, blue (primary) and orange (immortalized) crosses and final position, blue and orange crosses of the tracked primary and immortalized cell, respectively. The total distance traveled for selected cells were 606 μm for the primary cell and 207 μm for the immortalized cell. **(B)** Analysis of the speed of primary and immortalized cells at different passages. The speed of primary cells at passage 5, and of immortalized cells at both passage 5 and 17, decreased significantly. **(C)** Analysis of the distance covered by primary and immortalized whale Müller cells at different passages. Primary Müller cells at passage 5, and immortalized Müller cells at passage 5 and 17, covered significantly shorter distances. Scale bar: 50 μm. *p-value < 0.05.

Primary and immortalized Müller cells were tracked for 9 h, analyzing the speed and the distance traveled of at least 20 cells in each culture. The speed of Müller cells in primary cultures dropped from passage 0 (121.07 ± 9.39 μm/min), to passage 5 (70.80 ± 7.31 μm/min) or passage 8 (91.58 ± 10.51 μm/min). By contrast, the speed of immortalized Müller cells increased slightly from passage 5 (36.48 ± 2.74 μm/min) to passage 17 (51.87 ± 5.06 μm/min: Table 4). The distance covered by Müller cells was also measured over the 9 h period in culture, that of primary Müller cells falling from 1,210.65 ± 93.93 μm at passage 0 to 707.96 ± 73.14 μm at passage 5 or 915.77 ± 105.11 μm at passage 8. By contrast, immortalized Müller cells traveled shorter distances at passage 5 (364.78 ± 27.44 μm) and passage 8 (518 ± 50.58 μm: Table 4). Surprisingly, the speed and distance traveled of both primary and immortalized Müller cells decreased at later passages relative to those at passage 0, with more pronounced changes for immortalized Müller cells (Figure 7).

**TABLE 4:** Speed and distance of primary and immortalized whale Müller cells in culture.

Primary cells	Speed (μm/min)	Immortalized	Speed (μm/min)
Passage 0	121.07 ± 9.39	–	–
Passage 5	70.80 ± 7.31	Passage 5	36.48 ± 2.74
Passage 8	91.58 ± 10.51	Passage 17	51.87 ± 5.06

**Primary cells**	**Distance (μm)**	**Immortalized**	**Distance (μm)**

Passage 0	1,210.65 ± 93.93	–	–
Passage 5	707.96 ± 73.14	Passage 5	364.78 ± 27.44
Passage 8	915.77 ± 105.11	Passage 17	518.68 ± 50.58

### Characterization of Primary and Immortalized Whale Müller Cells

**FIGURE 8:**
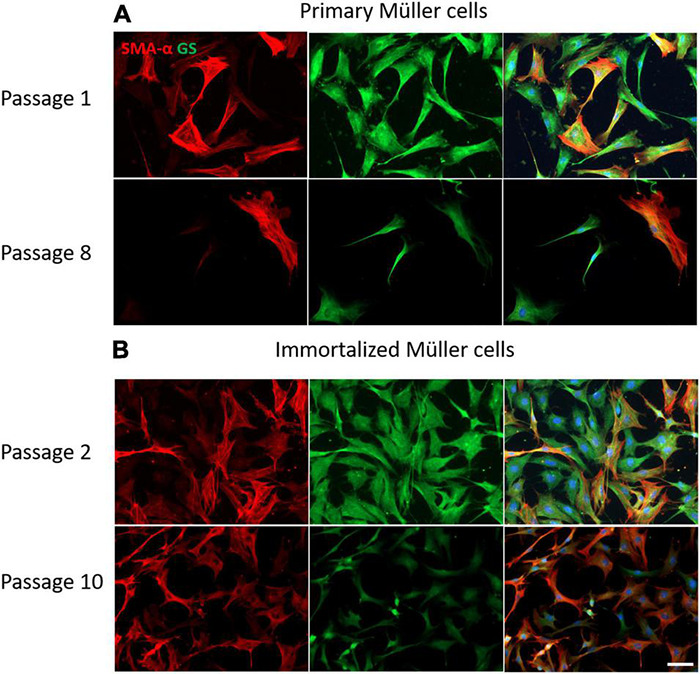
Expression of α-SMA (red) and GS (green) in cultures of primary and immortalized whale Müller cells at different passages. **(A)** Images of primary Müller cells from passage 1 and 8 of the cultures. Note that only some of the cells express α-SMA at passage 1 and 8, while the expression of GS persisted at both passages. **(B)** Images of immortalized Müller cells from passage 2 and 10 of the cultures. Note that at passage 2 only some cells expressed α-SMA, whereas virtually all the cells express α-SMA at passage 10 and the expression of GS was weaker. The nuclei of the cells were stained with DAPI (blue). Scale bar: 50 μm.

The expression of Müller cell molecular markers was analyzed in the primary and immortalized whale cell cultures at different passages, specifically that of GS and GFAP, as well as that of the neural marker β-III-Tubulin and the dedifferentiation marker α-smooth muscle actin (α–SMA). The expression of GS by cells in primary cultures persisted up to passage 8 when the Müller cells became senescent and in immortalized Müller cells the intensity of GS expression diminished at passage 10. While α-SMA is a dedifferentiation marker expressed strongly by fibroblasts, there was some heterogeneity in the expression of this marker in both primary and immortalized Müller cells at early passages, with some cells expressing α-SMA and others not. This heterogeneity persisted in primary Müller cells at passage 8, yet almost all the immortalized cells expressed α-SMA at passage 10 (Figure 8).

**FIGURE 9:**
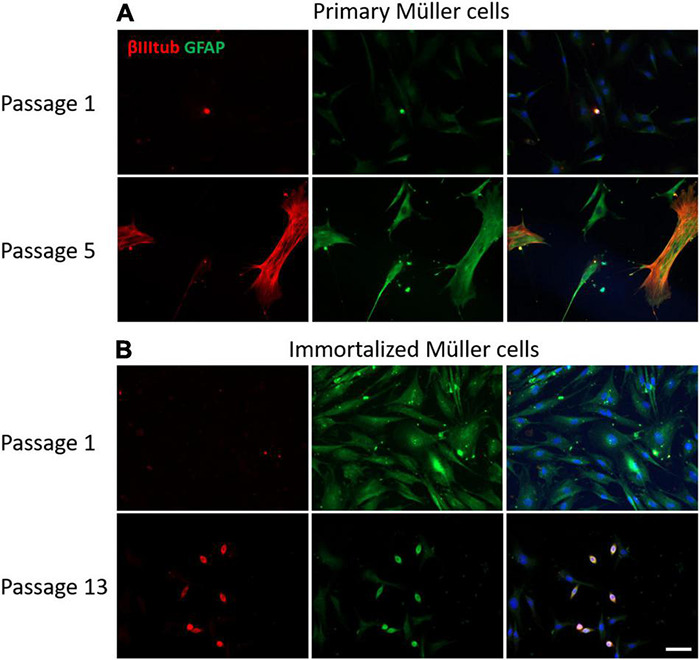
Expression of β-III-tubulin (red) and GFAP (green) in cultures of primary and immortalized Müller cells at different passages. **(A)** Images of primary Müller cells from passages 1 and 5 of the cultures. Note that the expression of β-III-tubulin increased at passage 5 of the primary Müller cell cultures, a point at which GFAP expression also increased. **(B)** Images of immortalized Müller cells from passages 1 and 13 of the cultures. While β-III-tubulin was not expressed at passage 1, it was expressed by rounded cells in the cultures at passage 13. GFAP was expressed by immortalized cells at passage 1 and only by rounded cells in passage 13. The nucleus of the cells was stained with DAPI (blue). Scale bar: 50 μm.

The GFAP is an indicator of tissue stress and it was expressed weakly at early passages of the primary cultures, increasing at passage 8. By contrast, GFAP was expressed strongly by immortalized Müller cells at early passages, decreasing at passage 10. As expected, the neural marker β-III-Tubulin was not expressed in the early passages of either primary or immortalized Müller cells, yet surprisingly the expression of β-III-Tubulin increased in advanced passages of the primary Müller cells. Interestingly, β-III-Tubulin expression was only enhanced in those immortalized cells that underwent a morphological shift toward a round shape (Figure 9).

### Morphological Heterogeneity of Immortalized Whale Müller Cells

**FIGURE 10:**
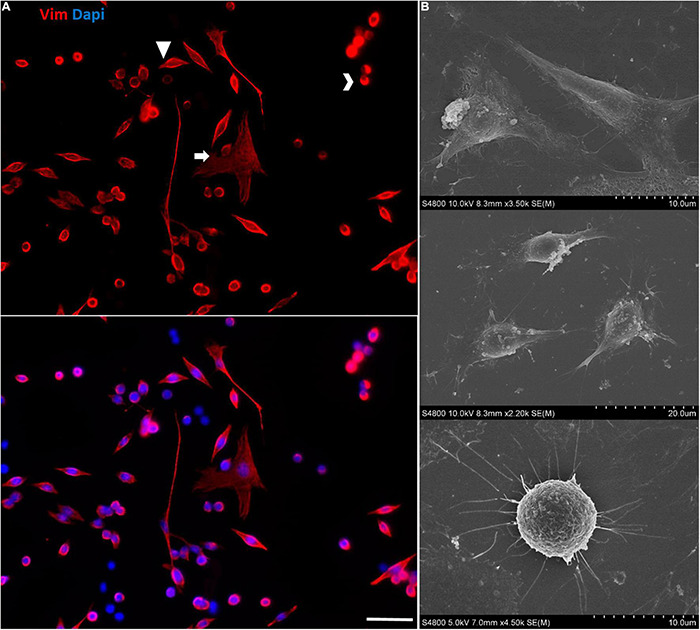
Morphological heterogeneity in the cultures of immortalized Müller cells at later passages. **(A)** Note the different morphologies of the immortalized Müller cells at passage 25, mainly large flat elongated cells and small round cells. The cells were labeled with antibodies against Vimentin (red) and their nuclei were stained with DAPI (blue). Scale bar: 50 μm. **(B)** SEM images of the morphologically distinct cells in the immortalized Müller cell cultures.

At later passages of the immortalized Müller cells, different cell morphologies were detected in the cultures and rather than the large, flat, elongated shape of the cells at early passages, some cells adopted a round shape (Figure 10).

## Discussion

In the present study, the adult Müller glia of one of the world’s largest mammals, the Sei whale, was analyzed *in vitro*. The aim of this work was to evaluate the characteristics of Müller cells and in an attempt to overcome the difficulties in obtaining cetacean material for such analyses, whale Müller cells were immortalized. Thus, we were able to analyze both primary and immortalized whale Müller glia.

Studying the viability of the whale Müller glia revealed that primary cells enter senescence at passage 8 while the immortalized cells continue to grow at least until passage 24 (Figure 3). The number of passages before reaching senescence of Müller glia from other animals were not dissimilar (Table 5), ranging from passage 6-10 in species such as mouse, pig, human and whale, irrespective of the longevity or body size of each species (Yegorov and Zelenin, 2003). However, variability in the passages before entering senescence was found in different cell-types, with human fibroblasts entering senescence at 20 passages (Nogueira et al., 2021), human epithelial amniocytes entering senescence after only 3 passages (Walen, 2004) and human Müller cells entering senescence at passage 6 (Giannelli et al., 2011), similar to whale Müller glia. This relatively early entry into senescence could be a result of stress induced by the culture conditions used (Liu et al., 2017).

**TABLE 5:** Comparison of Müller cell proliferation kinetics in different species.

Animal	Lifespan (years)	Animal Size (kg)	Time between divisions (hours)	Passages prior to senescence	References
Mouse	3.5	0.02	13	6	Pereiro et al., 2020
Rat	4.2	0.2	12.40	5	Pereiro et al., 2020
Pig	27	60	12.77	10	Pereiro et al., 2020
Human	72.6	62.5	20.6* (HeLa cells)	6	Giannelli et al., 2011; *Sigoillot et al., 2011
Whale	74*	20,000	23,15	8	Present study *(Tacutu et al., 2018)

As the whale Müller cells were successfully immortalized, their karyotype, proliferation kinetics, cell death, motility and molecular markers were compared to those of primary cultures of Müller cells. The karyotype of the immortalized Müller cells was stable, with 2*n* = 44 chromosomes, in accordance with that of Sei whale fibroblasts in culture (Hsu and Benirschke, 1975) and other whale species (Burkard et al., 2015). As these karyotypes were obtained at passage 12, immortalization and relatively long-term culture did not appear to cause obvious cytogenetic abnormalities.

In terms of proliferation kinetics, the time between divisions of primary whale Müller cells is around 24 h and it remains consistent across the passages (Figure 4). Previous studies on primary adult Müller cells from pig, rat and mouse showed the time between divisions to be approximately 12 h (Pereiro et al., 2020), around half the time taken by the whale primary Müller cells (Table 5). These differences in the time between divisions could be explained by the size of the animal, as smaller species have higher rates of cell metabolism and they are able to process resources at a faster rate than large species (Tourmente and Roldan, 2015). However, the time between divisions in immortalized Müller cells increases significantly to around 28 h, a delay that took place immediately after immortalization and persisted at later passages. This finding was unexpected and as far as we know, no similar changes in the time between divisions have been associated with immortalization. However, the transduction efficiencies of T-antigen-encoding vectors may be low and its expression may be transient, and therefore, transformation associated with inconsistent delivery and/or expression could produce genomic variability and morphological changes (Mayne et al., 1986; Ouellette et al., 2000), which could explain the increase in time between divisions of immortalized cells.

The time that Müller cells take to divide does not change after immortalization or with time in culture (Figure 4). However, the detailed morphological study of the events that take place during cell division in culture revealed an alteration in the formation of the cleavage furrow at later passages that is a consequence of the contractile ring (Alberts et al., 2002). Previous studies showed that p38α mitogen-activated protein kinase (MAPK) deficiency induces actin disassembly upon aging and a failure of cytokinesis that leads to enhanced binucleation (Bakin et al., 2004). Therefore, we cannot rule out that such is the case in cultured immortalized Müller cells due to altered cytokinesis, which could alter the aging process in culture, as well as delay the time between divisions.

As expected, the percentage of dividing cells increases in immortalized Müller cell cultures at later passages relative to primary cell cultures (Figure 5). There is a trend toward an increase in the proportion of immortalized cells that divide, which could be due to passage selection. Subculture of the viral transduced cultures progressively selects immortalized cells and thus, while at passage 5 the cultures contain a mixture of immortalized and non-immortalized (primary) cells, at passage 17 all the cells would be expected to be immortalized. This gradual change in the nature of the cultures may make it difficult to observe differences in the percentage of dividing cells. In primary cultures, only a subset (25%) of human Müller cells proliferate actively (Giannelli et al., 2011), more than in rat and mouse primary Müller cells that are around 15% (Pereiro et al., 2020), suggesting differences in the ability to re-enter the cell cycle after plating or due to heterogeneity in the Müller cells, as proposed previously (Vecino et al., 2016). In other immortalizations of Müller glia, such as Rat TR-MUL cells immortalized *in vitro*, the doubling time increased rapidly (Tomi et al., 2003), as occurred with other cell types like spontaneously immortalized epithelial cells from normal human conjunctiva in which proliferative cells (Ki67 positive cells) increased with the passages (Diebold et al., 2003).

The percentage of immortalized Müller cell death was low and did not change with the passages of the cells (Figure 6). It is known that SV40 Tag downregulates Rb and p53, proteins involved in apoptosis (Morgenbesser et al., 1994). By contrast, cell death in primary cultures of whale Müller cells increased significantly at passage 5, not surprising as the lifespan of primary cells is limited to several passages and the program of senescence is characterized by lysosomal activity, mitochondrial dysfunction, nuclear changes, chromatin rearrangements, endoplasmic reticulum stress and DNA damage, in association with a senescence-associated secretory phenotype (Sharpless and Sherr, 2015). Therefore, transduction of these cells with SV40 Tag maintains their replicative capacity, avoiding growth arrest, apoptosis and senescence.

The motility of the primary and immortalized whale Müller cells was assessed by analyzing their speed and the distance they covered in time-lapse videos (Figure 7). These cells covered the largest distances most rapidly at passage 0, parameters that decrease significantly at later passages, particularly in immortalized cells. Moreover, there were morphological changes to immortalized whale cells at later passages, shifting from more flattened and elongated cells to a round shape (Figure 10). These changes are associated with altered motility, and modifications in cell-cell and cell-substrate adhesion, as witnessed in epithelial cell lines (Matthay et al., 1993). Cytoskeletal changes during endothelial cell immortalization have been associated with the repression of cyclin-dependent kinase inhibitor 2A (p16INK4a), inducing random motility (Kan et al., 2012). Other immortalized cell lines like human periodontal ligament fibroblast (hPLF-hTERT) also underwent changes related to immortalization that specifically affect cell migration, cell motility and cell adhesion (Nogueira et al., 2021). Hence, the changes to the immortalized Müller cells witnessed here could be characteristic of the process of immortalization.

Whale primary and immortalized Müller cells were also characterized immunocytochemically using the Müller cells markers GS (Mack et al., 1998) and GFAP (Lewis et al., 1988), the neural marker β-III-Tubulin (Jiang et al., 2015) and the marker of dedifferentiation or a fibroblastic phenotype, α-SMA (Darby et al., 1990) (Figures 8, 9). Parallel studies demonstrated that these specific markers are present in the Müller glia of the whale retina (Ruzafa et al., 2022). GS is the main enzyme involved in transmitter recycling in Müller cells, and it is downregulated in immortalized Müller cells after 10 passages. However, the expression of GS persists in primary cultures even up to passage 8. In other species like pig, rat and mouse GS is also expressed by Müller cells in primary culture (Pereiro et al., 2020), although GS expression is strongly downregulated in cultured pig Müller cells after 8 days *in vitro* and only a small population of the cells remain GS immunoreactive (Hauck et al., 2003).

Heterogeneity among glial cells is widely known (Luna et al., 2010; Vecino et al., 2016) and hence, not all Müller cells in a retina may respond to a pathogenic stimulus in identical manner. Indeed, these cells even express proteins like GFAP heterogeneously, possibly due to the distinct roles they fulfill in the retina (Bringmann et al., 2006). It is known that in the first days of Müller cell culture they increase their GFAP expression, which thereafter diminishes as the culture progresses (Guidry, 1996). However, some Müller cells do maintain GFAP expression, whereas other cytoskeletal markers like vimentin are expressed homogeneously by Müller cells. GFAP is not expressed at passage 1 of whale Müller cell primary cultures but its expression is induced at passage 5. However, whale Müller cells increase GFAP expression immediately after immortalization at passage 1, whereas at passage 13 only the small rounded cells in the cultures express GFAP, confirming the heterogeneity in the present immortalized cell line. GFAP expression is sometimes induced by procedures that promote differentiation (Evrard et al., 1986), which could explain the increase in the expression of GFAP in the cells that change their morphology in the immortalized cultures.

Müller cells can rapidly change their protein expression *in vitro* (Hauck et al., 2003) and adopt a fibroblast-like phenotype (Fischer and Reh, 2003). Isolated porcine Muller cells in culture undergo phenotypic dedifferentiation to fibroblast-like cells and they acquire α-SMA expression (Guidry, 1996). A subpopulation of both primary and immortalized whale Müller cells express α-SMA *in vitro*, with all cells expressing α-SMA at later passages.

The expression of β-III-Tubulin increased in primary cell cultures at passage 5 and it is also expressed in the round cells that appeared in the immortalized whale Müller cell cultures. Previous studies of other Müller cell lines also reported the induction of β-III-Tubulin (Gomez-Vicente et al., 2013), suggesting that Müller cells dedifferentiate in both primary and immortalized cultures, as reflected by an increase in the expression of neural markers like β-III-Tubulin. The Mio-M1 cell line also expressed neural stem cell markers, including β-III tubulin, Sox2, Pax6, Chx10, and Notch 1 (Lawrence et al., 2007), and such changes in culture suggest these cells may potentially be used for cell-based therapies to restore retinal function. Furthermore, both β-III-Tubulin and α-SMA are proteins that may be expressed by Müller cells after cell damage. For example, β-III-Tubulin is overexpressed by Müller cells following retinal detachment (Lewis et al., 1995) and α-SMA expression is dramatically increased in retinas from humans with diabetic retinopathy (Zhou et al., 2017). These changes are remarkably similar to the phenotypic changes described following massive gliosis.

In summary, we describe here the establishment and characterization of a Whale 2021 Müller (W21M) cell line, which to our knowledge is the first immortalized cell line derived from Sei whale Müller cells. W21M cells display a heterogeneous cell morphology, less motility and a distinctive expression of some typical molecular markers of Müller cells, with an increase in dedifferentiation markers like α-SMA and β-III tubulin, while they preserve their GS expression depending on the culture passage. These immortalized cells proliferate more rapidly and they have a stable karyotype. As a result, the new transformed cell line described here cannot only be used in marine mammal research that can provide a unique opportunity for marine conservation research, extending to toxicological, bacteriological, virological and epidemiological studies. Also, it represents a unique tool to study key features of the interactions between Müller glia and neurons, contributing to the study of neuroprotection and neurogenesis, and to obtain detailed information about the characteristics of an important glia cell type in the retina of one of the largest mammals in the world.

## Data Availability Statement

The original contributions presented in the study are included in the article/Supplementary Material, further inquiries can be directed to the corresponding authors.

## Ethics Statement

Ethical review and approval was not required for the animal study because the retina of *Balaenoptera borealis* was obtained from a beached specimen with the regional authorities permission.

## Author Contributions

XP and EV conceived and designed the experiments. XP, SB, and LR contributed in the data analysis and wrote the manuscript. XP and SB contributed in the imaging tools. XP, EV, NR, and DR-V contributed to manuscript revision and read, and approved the submitted version. All authors contributed to the article and approved the submitted version.

## Conflict of Interest

The authors declare that the research was conducted in the absence of any commercial or financial relationships that could be construed as a potential conflict of interest.

## Publisher’s Note

All claims expressed in this article are solely those of the authors and do not necessarily represent those of their affiliated organizations, or those of the publisher, the editors and the reviewers. Any product that may be evaluated in this article, or claim that may be made by its manufacturer, is not guaranteed or endorsed by the publisher.
