# Supplementary material for: Characteristics of Whale Müller Glia in Primary and Immortalized Cultures
Source: Front Neurosci. 2022 Mar 14;16:854278. doi: 10.3389/fnins.2022.854278 (PMC8964101; doi:10.3389/fnins.2022.854278)
Supplement: Supplementary file 1 [file Table_1.DOCX]

| Antigen | Host | Supplier (ref) | RRID |
| --- | --- | --- | --- |
| β-III-Tubulin | Mouse | Abcam (ab7751) | AB_306045 |
| Glial fibrillary acidic protein (GFAP) | Rabbit | Sigma-Aldrich (G9269) | AB_477035 |
| Glutamine synthetase (GS) | Rabbit | Abcam (ab49873) | AB_880241 |
| Smooth muscle actin-α (α-SMA) | Mouse | Abcam (ab7817) | AB_262054 |
| Vimentin | Rabbit | Abcam (ab92547) | AB_10562134 |

Suppl. Table 1. Primary antibodies all used at a working dilution of 1:1,000
