# Supplementary material for: Characteristics of Whale Müller Glia in Primary and Immortalized Cultures
Source: Front Neurosci. 2022 Mar 14;16:854278. doi: 10.3389/fnins.2022.854278 (PMC8964101; doi:10.3389/fnins.2022.854278)
Supplement: Supplementary file 2 [file Data_Sheet_1.docx]

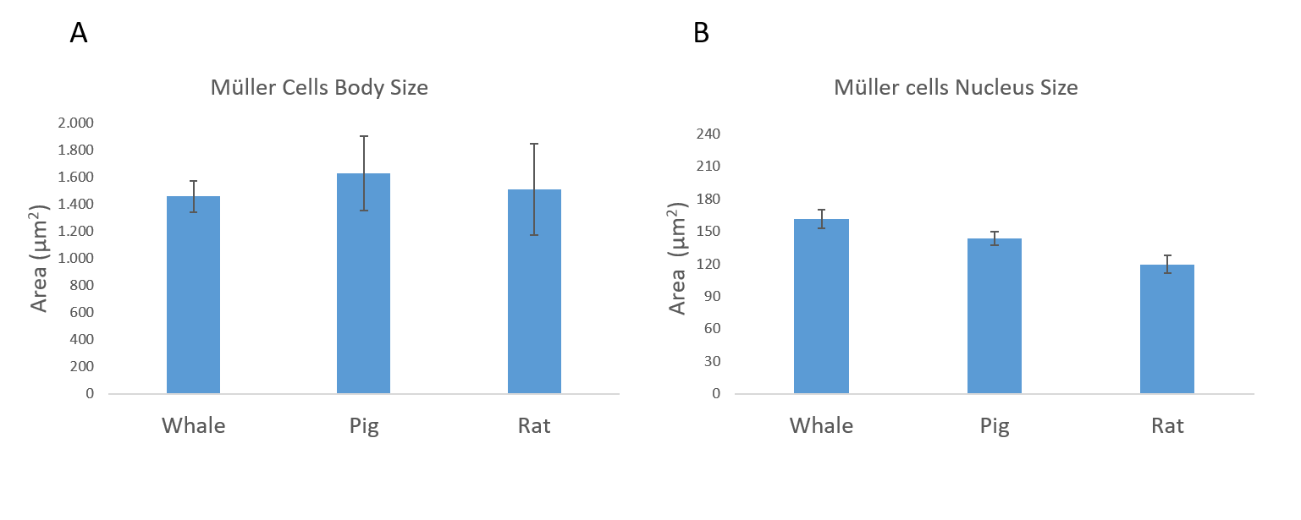


**Suppl. Figure 1. Analysis of the body and nucleus size from primary whale Müller cells compared to pig and rat Müller cells.** (A) Area (µm^2^) of the cell body from whale, pig and rat Müller cells. (B) Area (µm^2^) of the nucleus from whale, pig and rat Müller cells. No differences were found, one-way ANOVA, followed by Bonferroni test. (p-value>0.05).
